# Mobile health technology, exercise adherence and optimal nutrition post rehabilitation among people with Parkinson’s Disease (mHEXANUT) – a randomized controlled trial protocol

**DOI:** 10.1186/s12883-023-03134-5

**Published:** 2023-03-02

**Authors:** Sigrid Ryeng Alnes, Ellisiv Lærum-Onsager, Asta Bye, Annette Vistven, Erika Franzén, Mette Holst, Therese Brovold

**Affiliations:** 1 Department of Rehabilitation Science and Health Technology, Faculty of Health Science, OsloMet - Oslo Metropolitan University, Oslo, Norway; 2 Department of Nursing, Lovisenberg Diaconal University College, Oslo, Norway; 3 Department of Behavioural Sciences in Medicine, Faculty of Medicine, University of Oslo, Oslo, Norway; 4 Department of Nursing and Health Promotion, Faculty of Health Science, OsloMet - Oslo Metropolitan University, Oslo, Norway; 5 European Palliative Care Research Centre, Department of Oncology, Oslo University Hospital, Oslo, Norway; 6 Institute of Clinical Medicine, University of Oslo, Oslo, Norway; 7 Unicare Fram Rehabilitation Centre, Rykkin, Norway; 8 Division of Physiotherapy, Department of Neurobiology, Care Sciences and Society, Karolinska Institutet, Stockholm, Sweden; 9 Medical Unit Occupational Therapy & Physical Therapy, Theme Women’s Health and Allied Health Professionals, Karolinska University Hospital, Stockholm, Sweden; 10 Nutrition Research Unit, Center for Nutrition and Intestinal Failure, Aalborg University Hospital, Aalborg, Denmark; 11 Department of Clinical Medicine, Aalborg University, Aalborg, Denmark

**Keywords:** Parkinson’s Disease, Digital health, m-health, Exercise, Nutrition, Activity tracker

## Abstract

**Background:**

Although it is well known that regular physical activity and exercise, as well as maintaining adequate nutritional status is important to delaying symptom development and maintaining physical capacity and function in people with Parkinson’s Disease (PD), many are unable to follow self-management recommendations. Active interventions have shown short-term effects, but there is a need for interventions that facilitate self-management over the course of the disease. Until now, no studies have combined exercise and nutritional interventions with an individual self-management approach in PD. Thus, we aim to examine the effect of a six-month mobile health technology(m-health)-based follow-up programme, focusing on self-management in exercise and nutrition, after an in-service interdisciplinary rehabilitation programme.

**Methods:**

A single-blinded, two-group randomised controlled trial. Participants are Adults aged 40 or older, with idiopathic PD, Hoehn and Yahr 1–3, living at home. The intervention group receives a monthly, individualized, digital conversation with a PT, combined with use of an activity tracker. People at nutritional risk get additional digital-follow-up from a nutritional specialist. The control group receives usual care. The primary outcome is physical capacity, measured by 6-min walk test (6MWT). Secondary outcomes are nutritional status, Health related quality of life (HRQOL), physical function and exercise adherence. All measurements are performed at baseline, after 3 months and after 6 months. Sample size, based on primary outcome, is set at 100 participants randomized into the two arms, including an estimated 20% drop out.

**Discussion:**

The increasing prevalence of PD globally makes it even more important to develop evidence-based interventions that can increase motivation to stay active, promote adequate nutritional status and improve self-management in people with PD. The individually tailored digital follow-up programme, based on evidence-based practice, has the potential to promote evidence-based decision-making and to empower people with PD to implement exercise and optimal nutrition in their daily lives and, hopefully, increase adherence to exercise and nutritional recommendations.

**Trial registration:**

ClinicalTrials.gov (NCT04945876). First registration 01.03.2021.

**Supplementary Information:**

The online version contains supplementary material available at 10.1186/s12883-023-03134-5.

## Background

Parkinson’s disease (PD) is the fastest growing neurological disorder in the world [1]. It is a progressive and disabling disease with both motor and non-motor symptoms [2], potentially affecting several domains of a person’s life, including nutritional status [3, 4], health-related quality of life (HRQOL) [5], physical function and activity for daily living [6, 7].

It is known that physical activity and exercise can slow disease process and brain atrophy in PD, as well as delay symptom development [8], improve cognitive and physical function and capacity [9–12], and increase HRQOL [12–14]. Physical activity and exercise are closely related constructs but have different meanings, where the former is any bodily movement produced by the skeletal muscles resulting in energy expenditure, while the latter is intentional physical activity for improving health and fitness [15]. Due to the well-known effects of physical activity and exercise, they are key elements in today’s treatment guidelines and a combination of regular physical activity and exercise of moderate to high intensity is recommended [16]. Multiple forms of exercise are effective [8, 12, 17, 18], but have primarily short-term effect [19], and after a supervised exercise period, measures tend to return toward baseline values [12, 17, 20]. This regression may partly reflect the progressive nature of PD, but previous research suggests that maintaining motivation for exercise is difficult once formalised interventions end [20–24]. Experiences of poor health and disability symptoms may contribute to self-imposed activity restrictions and inactivity [25]. In addition, the experience of apathy, affecting the ability to initiate exercise, is a common non-motor symptom in PD [26]. Knowing the benefits of physical activity and exercise is not enough to change from a sedentary to an active lifestyle [27] and many people with PD remain sedentary [18, 28, 29].

Good nutritional status is an essential predictor of health status among people with PD [3], and unintentional weight loss is a commonly documented symptom [3, 30, 31]. Symptoms associated with PD [32] and pharmaceutical therapies used to alleviate PD symptoms [31] are both potential factors that can decrease food and energy intake, leading to weight loss. It has been reported that between 2 and 34% of people with PD— depending on population and measurement methods—are at risk of malnutrition [33, 34]. Malnutrition is associated with adverse outcomes such as increased mortality, hospitalisation, and reduced physical function [30]. Not having enough energy to fuel daily activity can lead to fatigue, decreased endurance and reduce overall physical function. Although previous studies have concluded that nutritional education can improve nutritional status in community-dwelling older adults [35, 36], there is limited research evidence on this topic in the PD population [37]. Nevertheless, the nutritional guidelines for people with PD focuses on early detection of nutritional problems, advice for common nutritional challenges and maintenance or improvement of physical function [38, 39]. In addition, optimal nutrition and dietary habits can positively affect the effectiveness of PD medications and potentially slow down disease development [40], as well as improve symptom management and overall well-being [39, 40].

Over the years, patients have been playing a greater role in managing their disease, and a key element of treatment is self-management [41–43]. Self-management refers to the different tasks an individual must take on in the day-to-day management of chronic conditions to live a decent life with the disease [44, 45]. Although previous research suggests that both exercise and nutrition should be part of long-term self-management in people with PD [3, 12, 42], adherence to self-management regimens tends to vary greatly [46, 47]. Research indicates that follow-up at home after rehabilitation is necessary to increase optimal nutrition and adherence to exercise [21, 48] and people with PD have identified self-management components as a research priority [49]. Consequently, the challenges for long-term self-management to recommendations are an important future concern for research and practice. To improve the management of PD in a community setting, people with PD, and their caregivers, have among other things expressed a desire for a person-and-community-centred approach, accessibility to support systems, information on health care services and a more comprehensive approach [50].

Several factors that can help increase adherence to nutrition and exercise recommendations among people with PD have been suggested [18, 47, 51]. Self-efficacy, an individual’s belief in their own ability to carry out an action successfully [23, 52], plays a key role in adherence to regular exercise [53]. Instructions and education are important, but not enough. Help to overcome barriers and identify motivators to engage in exercise is necessary [54], and both personal and environmental factors need to be addressed [55]. Wearable technology, such as activity trackers, is designed to motivate and offer support to individuals in self-monitoring and increasing their daily physical activity and exercise [56]. Such activity trackers can also monitor heart rate and exercise intensity [57], and thus facilitate a greater awareness of exercise at the recommended intensity level. They can promote physical activity among people with PD, though more research is necessary [58–63]. Further, social support is vital for many people with PD after rehabilitation [64–66] and physiotherapists (PTs) can play a crucial role in enabling people to improve their health, well-being and HQOL [67]. A large range of potential barriers and motivators for exercise in people with PD is found, and it is often experienced as difficult to achieve the recommended exercise dose outside clinical and research settings [54]. PTs are recognized as important in both identifying and addressing the person specific barriers and motivators, and through this, facilitate a better adherence to recommendations [54]. However, today, long-term PT follow-up among people with PD is often random or rare, despite being a priority [68].

Telehealth/m-health is found to be a potentially low-cost and efficient element within healthcare treatment programs [69, 70], and both patients and caregivers are generally positive to these forms of treatment [69–71]. This also includes people with PD who in one study responded that telehealth was convenient, easy to use, had the potential to give the same quality of follow-up care as in-person visits, and that telehealth could make follow-up easier in cases were traveling was problematic [71]. Even though the transition to more digital solutions was already established, the recent pandemic has accelerated the development of digital health care [71–74], increasing the relevance of these types of interventions. A monthly digital follow-up on physical activity, exercise, and nutrition, might make long-term follow-up more accessible and less time-consuming for individuals, potentially reducing costs to society [69] and hopefully improving long-term self-management in people with PD.

The combination of nutrition and exercise has been shown to improve health outcomes, and interventions combining both is found to have better effects than either one alone in other populations [75–77]. This combination has not yet been studied in the PD population, but it is suggested that it has the potential to slow down the progression of the disease [40]. A focus on self-management with a comprehensive approach is both desired [50, 78] and recommended for future research in this area [49, 79].

Based on current knowledge, we have designed a new m-health follow-up programme to improve self-management of nutrition and physical activity among people with PD after an in-service interdisciplinary rehabilitation programme at a rehabilitation centre in Norway. The follow-up programme includes a monthly, individualised digital conversation (video or telephone) on, e.g. motivation, exercise, nutrition, and other relevant areas, combined with the use of an activity tracker. People at nutritional risk will receive additional digital follow-up from a registered nurse (RN) specialised in nutrition. To our knowledge, no study has examined the effect of an m-health follow-up programme focusing on physical activity, exercise, nutritional status, and HRQOL among people with PD. Therefore, the objectives of this study are:

1. To examine the effect of an individualised, m-health follow-up programme focusing on self-management in physical activity, exercise, and nutrition on physical capacity among people with PD after attending an in-service interdisciplinary rehabilitation programme.
2. Secondary objectives are to examine the effects of the m-health follow-up programme on nutritional status, HRQOL, physical function and exercise adherence.

## Methods

### Design

This is a single-blinded, two-group randomised controlled trial. Participants are recruited after attending a 4–5-week interdisciplinary rehabilitation programme at a rehabilitation centre. The intervention group will receive six-month of individually tailored m-health-based follow-up focusing on self-management of exercise and nutrition, and use of an activity tracker for motivation and monitoring activity levels. The control group will receive usual care, except for re-testing.

### Participants

**Fig. 1:**
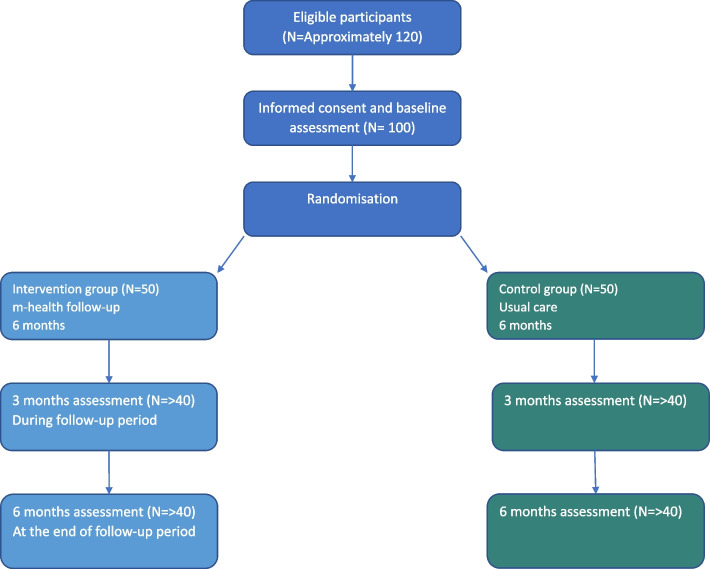
Planned Flow of Participants

Participants will be recruited during their elective admission stay at the rehabilitation centre. Eligible participants will be contacted by the research assistant (PT) and given oral and written information about the study. All participants will provide written informed consent before participating in the study. See Fig. 1 for planned flow of participants.

### Inclusion/exclusion criteria

Eligibility criteria were adults aged over 40 years, with a diagnosis of idiopathic PD and a Hoehn and Yahr stage of 1–3, living at home, within a maximum travel time of 2.5 h from the rehabilitation centre and in ownership of a smartphone.

The exclusion criteria were people with Hoehn and Yahr stage of 4–5 and medical issues that might affect participation in an exercise programme, as well as a dementia diagnose or severe dysphagia. People receiving enteral or parenteral nutrition and patients who, before admission to the rehabilitation centre exercised structured/planned, regularly more than twice weekly.

### Randomisation

The participants will be randomly assigned at a 1:1 ratio to the intervention or control group using a computer-generated, permuted block randomisation scheme, set up by an independent statistician before the trial. The PT responsible for the follow-up is getting the information on allocation on an ongoing basis from the principal investigator and informs the participants on which group they are allocated to after baseline testing, at the end of stay.

### Assessment and blinding

Baseline assessment will be conducted at the end of stay at the rehabilitation centre by a trained assessor. After three and six months (± one week), follow-up testing will be conducted at the rehabilitation centre by the same assessor. To increase accuracy, we will strive to conduct measurements at approximately the same time of day at baseline, three months, and six months. This to ensure that the participants are at the same medication level and in their on-phase, as this can affect function.

The assessor will be blinded to group allocation. Participants are asked not to share information on group allocation during assessments and, when applicable, to remove their activity tracker before meeting the assessor. Because of the nature of the intervention, we are unable to blind the treating therapist or the participants to group allocation. Participants are included at the end of stay so that they have limited opportunities to discuss their group allocation with other potential participants.

### Interdisciplinary rehabilitation programme at the rehabilitation centre

All participants are recruited after attending a 4–5-week rehabilitation programme at a rehabilitation centre in Norway. This is an individualized comprehensive interdisciplinary rehabilitation programme with focus on self-management of the disease including exercise and nutrition, based on European guidelines for treatment of people with PD [42]. The participants receive education on relevant PD-related topics, such as medication, nutrition, and exercise, combined with group-based and individual follow-up on, e.g. exercise, speech and language therapy, medication, and nutrition. At the end of stay they receive recommendations from their team at the centre on a home-based rehabilitation plan, focusing on areas relevant to the individual patient, as aforementioned. In this plan, all are encouraged to engage in cardiovascular exercise combined with symptomatic exercise, e.g. Parkinson wellness recovery, which often includes strength exercises. See appendix 1 for a full description of the intervention at the rehabilitation centre.

### The intervention

The current study evaluates the effect of a m-health-based follow-up programme after discharge from the rehabilitation centre. See Table 1 for intervention overview.

**Table 1:** The m-HEXANUT intervention programme

m-health-follow-up group	Usual care group
Conversation with the project PT:	Conversation with the project PT:
- Information on group allocation and re-testing, the nature of the digital follow-up, and education on use of the activity tracker	- Information on group allocation and re-testing
- Participants are encouraged to follow the home-based rehabilitation plan when they return home	- Participants are encouraged to follow the home-based rehabilitation plan when they return home
Digital start up call from the PT: focus on goals and motivation	No further project follow-up except for testing at three and six months
Monthly digital conversations with the PT, via video or telephone	
Participants can send text messages in-between planned sessions	
Up to two hours with a nutritional nurse, if nutritional risk	
Testing at three and six months	

### Inclusion conversation

All participants will have a conversation with the PT responsible for the digital follow-up at the end of stay at the rehabilitation centre. In this session, they receive information on group allocation and re-testing, and they are encouraged to follow the recommended individualised home-based rehabilitation plan when they return home.

### m-health follow-up group: m-health follow-up post-stay at the rehabilitation centre

#### Information on follow-up and use of activity tracker

During the inclusion conversation the follow-up group will receive information on the nature of the digital follow up and education on how to use the activity tracker.

#### Monthly tele-follow-up by a PT

The first follow-up session will be conducted within two weeks of returning home. The session will focus on the participants’ experiences with and motivations for exercise and optimal nutrition, as well as their goals and plans for exercise and nutrition in the coming weeks and potential barriers or challenges, and how to address them. The participants will be asked to describe the recommended home-based rehabilitation plan from the rehabilitation centre, their plan to implement it at home and their motivations for doing so. The participants can also describe other relevant challenges they want to address, e.g. regarding sleep, pain, or medication.

After the first follow-up session, they will receive monthly follow-up with the PT via video or telephone for support and to address questions or thoughts on such relevant areas as exercise, nutrition, motivation, sleep, gastrointestinal dysfunctions, balance between activity and rest or new potential symptoms. The PT can discuss all topics related to living with PD, and give answers or advice based on existing guidelines or direct them towards who to contact when the PT cannot—or should not—give advice. The participants can also contact the PT by text message between planned sessions if they have questions.

The PT will collaborate with relevant health care personnel in the municipality, when necessary. Appropriate collaborators might include PTs, nurses, occupational therapists or physicians, as well as members of ParkinsonNet, a professional network for health care personnel with expertise in PD [80].

#### Individually tailored follow-up on nutrition

All participants receive PD-related nutritional education at the rehabilitation centre. The intervention group is also encouraged to read through the digital resource ‘*Matvett på nett*’, an e-learning course on nutrition for people with PD. Participants in the intervention group scoring ≥ 4 on the abridged Patient-Generated Subjective Global Assessment (aPG-SGA) [81] will be offered up to two hours of additional individualised digital guidance on nutrition from the RN, based on their aPG-SGA results and mapping of their dietary intake. The PT will address nutrition throughout the follow-up period, involving the RN as needed. Nutrition guidance will follow the ESPEN guideline in clinical nutrition in neurology [82] and the Norwegian Guidelines developed by the Norwegian Directorate of Health [83]. If participants score < 4 on the aPG-SGA but show signs of nutritional risk during the follow-up period, such as weight loss, changes in appetite, or new symptoms that could affect nutrition, the RN can be involved as needed.

#### Daily use of the Garmin Vivosmart 4

The activity tracker can facilitate daily activity and continued exercise at the recommended intensity level at home. Participants will be introduced to using the Garmin wristband for motivation, daily activity tracking (steps, distance travelled and intensity minutes), logging of specific exercises (cardio/strength) and controlling exercise intensity using the activity tracker’s heart rate (HR) monitor.

It is possible to set activity goals on the device, so the participants can check how far from their activity goals they are and receive congratulatory notifications when they reach their goals. The goal for daily steps is automatically set so it adjusts to the individual participant’s activity level. The activity tracker logs intensity minutes as moderate (70–80% of maximum HR) or vigorous (80–100% of maximum HR), with a pre-set goal of 150 intensity minutes a week. This is in line with the physiotherapy guideline recommendations for aerobic physical activity [16]. The goal is reached when either 150 min of moderate intensity or 75 min of vigorous intensity exercise is completed. It is also recommended that participants log all exercise sessions in the activity wristband, allowing them to analyse their exercise efforts and, if desired, discuss them with the PT in the tele-conversations.

### Usual care group—no further project follow-up post-stay at the rehabilitation centre

The participants are to follow usual care, meaning that upon returning home after the inclusion conversation, they will receive no further instructions, except for re-testing at three and six months. This means that some participants, on their own initiative, might seek treatment from PTs, speech and language therapists or other relevant practitioners, while others might not. We will ask participants on degree of weekly physical activity and exercise, and degree and type of PT follow-up at baseline and 6 months follow-up.

### Education of intervention deliverers

The digital follow-up is primarily delivered by a PT (SRA) with an MSc in physical therapy. Participants at nutritional risk will also receive follow-up from an RN (ELO) with a PhD. in nutrition. The trained assessor (AV), responsible for all participant testing, is a PT with an MSc in physical therapy. The digital follow-up conversations are based on a semi-structured manual (Appendix 2), developed by the research group.

### Outcome measures

#### Baseline characteristics

At baseline, information about the participants health and sociodemographic variables, such as age, gender, living arrangements, education level, previous falls, home care services, physical activity level, degree and type of follow-up from PTs if any, medication list, comorbidities, and time of PD diagnosis, is assessed. Several of these variables will also be registered at follow-up to be able to assess changes in e.g. weekly exercise and medications. Food, diet habits and meal frequency are measured by the ‘*Hva Spiser Du?*’ (‘What do you eat?’) questionnaire. To assess the participants’ cognitive function, the Montreal Cognitive Assessment (MoCa) test is used, widely applied to screen for mild cognitive impairment and dementia and validated for the PD population [84, 85].

### Primary outcome

Physical capacity will be measured at baseline, three months, and six months with the 6MWT, recommended for measuring physical capacity [80, 86]. Further the Borg scale (6–20 scale) is used to obtain a subjective rate of perceived exertion during the test [87, 88], while HR is measured with a pulse oximeter before test start, after test end, one minute after test end, and two minutes after test end. 6MWT is an easy test with a low patient’s burden and is chosen due to its responsiveness, good validity for measuring physical capacity in people with PD, and clinical utility [86].

### Secondary outcomes

The secondary outcome measurements are performed at baseline, three and six months.

#### Nutritional status

In this study, to identify risk of malnutrition, the aPG-SGA is used, a validated, patient-reported instrument considered a gold standard for nutritional assessment [89–94]. The aPG-SGA includes self-reported questions on weight, food intake, symptoms, and activity level [90], and the total score indicates the need for an intervention and type of intervention needed (0–1 = no intervention needed, 2–3 = patient and family education, 4–8 = intervention by a dietitian and, ≥ 9 = critical need for symptom managing) [81]. The aPG-SGA is not validated on people with PD, but because it assesses common symptoms that can affect nutritional status in people with PD, it is well accepted for this use [95].

Both height and weight will be measured, and Body Mass Index (BMI) is calculated and interpreted in accordance with the World Health Organisations weight classifications [96].

Body composition is measured using a bioelectrical impedance analysis (BIA), a simple and quick non-invasive method to estimate body fat and muscle mass [97], used to analyse nutritional status [97, 98].

Swallowing difficulties are assessed using the Radboud Oral Motor Inventory (ROMP) questionnaire for PD [99, 100], developed to assess three areas: swallowing, speech, and saliva control [100]. It is quick to use, validated and reliable [99, 100].

Grip strength is measured using a hand-held dynamometer (Saehan SH5001) and is employed as a marker of nutritional status, as muscle function reacts early to nutritional deprivation. It measures the isometric muscle strength of the hand and forearm, and maximum strength is calculated by the mean of three trials, on both sides [101]. It is found to be valid and relevant in assessing nutritional status [102].

#### HRQOL

HEQOL are measured using the Parkinson’s Disease Questionnaire(PDQ39) [103], a 39 items self-report on PD disease-specific HRQOL in the last month. It assesses how often a person experienced difficulties in eight different QOL dimensions [104]. PDQ39 is selected because it is the most-used disease-specific health questionnaire in PD [105], and it is recommended in the European guidelines [42].

#### Balance and functional mobility

Balance is measured using the Mini Balance Evaluation Systems Test (Mini BESTest) [106], a 14-item balance assessment that predicts fall risk and balance impairments, and measures changes over time [107]. The test is recommended for PD and has excellent test–retest reliability and validity to detect balance deficits [108, 109].

The Five Times Sit to Stand test (FTSST) is a quick and easy test to measure an individual’s ability to transition between sitting and standing and is a method to quantify functional lower mobility strength [110]. The person is asked to stand up and sit down five times as quickly as possible without using their arms. The test is recommended in the European guidelines for physiotherapy and PD, is responsive to changes over time [16] and has excellent test–retest reliability and interrater reliability [111].

### Additional measures

For the intervention group, we will collect data on step count and HR, as well as exercise amount and intensity, from the Garmin Vivosmart 4 activity tracker. 

### Process evaluation

We will conduct a process evaluation of the implementation of this RCT guided by the UK medical research council’s framework for developing and evaluating complex interventions [112]. To assess adherence and to what extent the intervention was delivered as intended, a third party (the RN) will attend 10–15% of the sessions for observation. The PT will keep a log of all sessions describing how the delivery was conducted (video or telephone), length of session and themes discussed. Adherence to the use of the activity wristband will be documented through logging how many who used the activity wristband, if they used it manually or only automatically, and reasons why some did not use it. Participants will also receive a questionnaire with two questions asking how useful they found the digital follow-up and the activity wristband, on a scale from 1(not useful) to 5(very useful), at the end of the six-month follow-up. We will use a mixed model’s approach when evaluating the implementation process. Recruitment rates and reasons for not participating will be documented, and we will assess to which degree the sample is generalisable to the targeted population.

### Sample size calculation

The primary outcome of the current study is the difference between groups in physical capacity, measured by the 6MWT. Previous studies among older adults have defined a substantial, meaningful change between groups in the 6MWT to be a mean (SD) of 50 (80) meters. This estimate requires 82 participants, 41 in each group, and a clinical trial with an independent comparison of two groups at a significance level of 0.05 and 80% power is assumed. Considering possible losses during follow-up, the sample will be expanded by 20% (20 participants). Thus, 100 participants will be included in total [113].

### Statistical analyses

Results of the RCT will be reported following the CONSORT statement [114]. All statistical analysis will be conducted using the latest version of the SPSS and according to the intention to-treat principle (ITT). The normality of the distributions will be examined graphically by histograms and Q-Q plots, and by comparing the mean with the median. Descriptive data will be reported for variables of interest and reported as number (percent), mean (standard deviation) or median (25, 75 interquartile) based on what is appropriate. Between group differences at follow-up at three and six months after baseline will be assessed using linear mixed models for repeated measurements with a subject-specific random intercept. *P*-values < 0.005 will be considered statistically significant and all tests will be two sided. Effect size will be calculated (Cohen’s d).

## Results

Enrolment began in March 2021. Due to the pandemic situation, there have been some delays, but recruitment is planned to finish around January 2023. The main contribution of this article is a detailed protocol of our intervention and study design.

## Discussion

The study’s objectives are to evaluate the effect of a six-month m-health follow-up programme on primary outcome, physical capacity, and secondary outcomes nutritional status, HRQOL, physical function and exercise adherence. We anticipate that the intervention described in this protocol will have a positive impact on physical capacity, measured by the 6MWT. We also expect a positive impact on the secondary outcome measures.

Considering the expected increase in the number of older people with chronic diseases, including PD, as well as the individual and social burden of PD [1], we would argue it is crucial and beneficial to shed light on interventions that could potentially motivate people with PD to stay active, promote an adequate nutritional status and improve self-management. The individually tailored digital follow-up programme, based on evidence-based practice, has the potential to promote evidence-based decision-making and empower people with PD to implement exercise and optimal nutrition in their daily lives [47]. Further it has the potential to map barriers to and motivators for exercise and optimal nutrition for the individual participant and monthly follow-up by a therapist can help participants use this knowledge actively to increase adherence to exercise and nutritional recommendations [43, 47].

If proven effective, this programme may be beneficial for therapeutic practice and the health care system, both in primary (municipality) and specialist health care working with rehabilitation, and it may improve collaboration between PTs working in primary and secondary health care [68, 115]. First, if we can increase the patient’s ability for self-management of PD through a digital follow-up and consequently decrease the need for one-on-one time with a therapist, more patients can be treated simultaneously. Second, if more people manage to follow recommendations at home, the effect of rehabilitation might last longer and help more people in maintaining QOL and decrease the need for potentially unnecessary re-admissions to rehabilitation institutions.

The interventions used in this study consist of potentially low-cost strategies, such as telehealth and activity trackers that are easy to implement in similar settings. The different elements of the intervention are based on previous research on potential barriers and motivators and how to address them [24, 47, 54]. We also consider it a strength that the intervention is based on easily accessed web-based resources and guidelines and that the intervention used is not disease-specific and could be easily implemented among older people with different chronic diseases. A weakness to be considered is the digital literacy demands of using digital solutions, which might be too high for parts of this population. We aim to remedy this by adopting a pragmatic approach, offering follow-up by phone for those who cannot manage video calls and lowering the demands using the activity tracker or, in some cases, allowing no use of the tool at all.

Overall, the results from this study can inform future clinical practice and research as it could provide a potentially low-cost and easy-to-implement intervention that can be used as a supplement to today’s treatment regimen and has the potential to increase its efficiency, making it highly relevant research.

## Supplementary Information

**Additional file 1.** The Rehabilitation program at the rehabilitation centre

**Additional file 2.** Manual for start-up conversation

## Data Availability

Not applicable.

## Abbreviations

PD: Parkinson’s Disease
m-health: Mobile health technology
6MWT: 6-Minute walk test
HRQOL: Health related quality of life
RN: Registered nurse
PT: Physiotherapist
aPG-SGA: Abridged patient-generated subjective global assessment
HR: Heart rate
MoCa: Montreal cognitive assessment
BMI: Body mass index
BIA: Bioelectrical impedance analysis
ROMP: Radboud oral motor inventory
PDQ39: Parkinson’s Disease questionnaire
Mini BESTest: Mini balance evaluation systems test
FTSST: Five times sit to stand test
SD: Standard deviation
REK South/East: Regional comittees for medical research ethics south/east norway
ITT: Intention-to-treat
TSD: Services for sensitive data
