# Supplementary material for: Mobile health technology, exercise adherence and optimal nutrition post rehabilitation among people with Parkinson’s Disease (mHEXANUT) – a randomized controlled trial protocol
Source: BMC Neurol. 2023 Mar 2;23:93. doi: 10.1186/s12883-023-03134-5 (PMC9979434; doi:10.1186/s12883-023-03134-5)
Supplement: Supplementary file 1 — Additional file 1. The Rehabilitation program at the rehabilitation centre [file 12883_2023_3134_MOESM1_ESM.pdf]

The Rehabilitation program at the rehabilitation centre

All participants in both groups will receive the rehabilitation program currently provided at Unicare Fram rehabilitation centre. This is an individualized comprehensive and interdisciplinary rehabilitation program with focus on self-management of the disease including exercise and nutrition.

Description of the nutritional program for PD at Unicare Fram the participants will receive:

1. Group-based educational session on nutritional recommendation for people with PD, based on a digital course" matvett på nett", provided from the Norwegian Parkinson Association, based on the European recommendation on nutrition and PD. 45 minutes with the research assistant.
2. Group-based educational session on general nutritional recommendation based on the recommendations from the Norwegian Directorate of Health and is aimed at all patients at Fram regardless of diagnose. 45 min with a nutritional therapist.
3. Group-based educational session on non-motor symptoms in PD patients based on European guidelines (60). 45 min with a Parkinson nurse.
4. Group-based educational session on dysphagia. 45 min with a speech and language therapist.
5. Fram model – a brochure (printed and digital) based on the guidelines on self-management on exercise and nutrition for people with PD.

The educational sessions on nutrition will include the following content: nutritional value of food, designing a healthy diet, including how to estimate individual daily energy intake, and how to prevent malnutrition and weight loss, and advice on dietary adaptation to address the most common nutritional problems in people with PD, such as protein redistribution and levodopa, diet therapy to prevent/treat constipation, oropharyngeal dysphagia, and adaptation of texture. It is primarily aimed at the patients, but next of kin will also be invited to participate in the educational sessions.

Description of the exercise program for PD at Unicare Fram that all participants will receive:

1. Group-based educational session on exercise for people with PD. The educational sessions will be based on EBP recommendations for exercise in people with PD presented earlier in this application and recommendation from The European Physiotherapy Guidelines for Parkinson's disease (61). The education sessions will include information about the benefits and the importance of regular physical exercise for physical function and health. 1 x 45 min with a physical therapist.
2. Access to participation in a variety of exercise groups, such as spinning and strength exercise, as well as a total of 3 hours individual exercise sessions a week with a physical therapist according to the patients' needs and goals.
3. During their stay at the rehabilitation centre, the participants will together with their physiotherapist, design an exercise program to continue to do at home. According to a recent systematic review as well as one Cochrane review, no consensus of best exercise interventions for people with PD does exists (4, 11) and it is recommended that the exercise program is individually tailored based on the participant's needs and function (25).

4. Fram-model – a brochure (printed and digital) based on the guidelines on self-management on exercise and nutrition for people with PD.

The educational sessions on physical exercise will in line with recent research, include a discussion on common barriers to physical exercise among people with PD, such as fear of falling, lack of perceived benefits of physical exercise and low perceived health status to increase adherence to exercise (24). Furthermore, the sessions will include how the participants can set personal goals and can incorporate exercise and physical activity into activities of daily living. All exercise programs are based on European physiotherapy guidelines for people with PD (60). In addition, patients were offered participation in various self-efficacy experiences through the rehabilitation stay such as outdoor climbing, hiking, golf, training in the pool, badminton, and table tennis.

Other PD related activities:

The neurologist has a consultation with the patients´ at the beginning of the stay and follow ups when needed. The neurologist has weekly group sessions on the topic medication and the disease in general. The participants can attend a group session on the topic "PD and non- motor symptoms" with a nurse, in addition to individual consultation if needed. The occupational therapist has an educational session on PD and daily living (ADL) and offers individual consultation if needed.

Link to the Rehabilitations centres own description of their program for Parkinson's Disease:

[FRAM MODELLEN (unicare.no)]
