# Supplementary material for: Mobile health technology, exercise adherence and optimal nutrition post rehabilitation among people with Parkinson’s Disease (mHEXANUT) – a randomized controlled trial protocol
Source: BMC Neurol. 2023 Mar 2;23:93. doi: 10.1186/s12883-023-03134-5 (PMC9979434; doi:10.1186/s12883-023-03134-5)
Supplement: Supplementary file 2 — Additional file 2. Manual for start-up conversation [file 12883_2023_3134_MOESM2_ESM.docx]

**Manual for start-up conversation**

Duration of call: Up to 60 min

Start with helping the participant setting up a video-call. If they are unable to do this, or this causes unnecessary stress the conversations can be done via telephone.

If they want to include next of kin in the conversations, they are free to do so.

General

The PT gives a repetition of the nature of the digital conversations. The primary focus of the conversations will be physical activity, exercise, and nutrition, but it is meant to be a resource conversation for the participant where all areas of living with PD can be discussed.

This is their conversation, and it is important to give them time to explain in their own words, but to ask follow-up questions when necessary.

The PT can ask questions, try to motivate, give advice, and answer potential questions, but the participants decide for themselves what they want to talk about and how they choose to follow-up on the things discussed in the conversations.

If they have any questions in between sessions, they can send a text message to the PT. The PT will follow-up on the text via phone (text or call) or in the next planned session depending on the nature of the question.

Exercise

The PT asks the participant to describe their past and present experience with, and motivation for, physical activity and exercise. The PT follows up, asking about their current plans and recommendations for weekly exercise. They are also asked if they have any specific goals regarding exercise, and if not are encouraged to think about potential goals. The PT can give tips for goalsetting and some examples, but without making the goals for them.

Exact questions to ask will vary; the goal of the conversation is that they come up with a concrete plan for weekly exercise that they feel is realistic and feasible. The individual needs, motivation and self-efficacy beliefs will vary, and the plan should be adjusted accordingly. The PT should not make the plan for them but give them support in making it themselves. The plan should be based on the recommendations from the rehabilitation centre but can be adjusted based on what resources the participants have available and their own thoughts on what promotes and inhibits motivation for exercise.

All participants should have a combination of cardiovascular exercise combined with strength and symptomatic exercise at the recommended intensity level, in line with the guidelines for physiotherapy for people with PD. Not all are able to implement all elements immediately after returning home and must do a gradually increase of exercise, and some might have troubles reaching the right intensity and need help to do so. The advice, exercise plan and goals must be individually adjusted. Some might just need small adjustments, others need to be encouraged to train with a PT, or in a group or get advice on different exercise methods to try out, to get their heart rate up.

Nutrition

All participants are encouraged to take the “Matvett på Nett”, an e-learning course on nutrition for people with PD.

The PT gives a short introduction on why we are focusing on nutrition and informs that we want to increase the awareness regarding nutrition, to increase the possibility for early detection of nutritional challenges and thus early intervention.

The participants are asked if they have any nutritional challenges today and if so, if they have any strategies in dealing with this, what they are and how they are working for them so far.

They are specifically asked about appetite, water intake, awareness around when they take medication in relation to meals and any habits to monitor weight change.

If the PT detect any nutritional challenges, it will be considered whether they are to be defined as being at nutritional risk. In all cases the PT will give advice on potential strategies to remedy the challenges they experience. This is based on guidelines for nutrition for people with Parkinson’s disease.

Those identified to be at nutritional risk, either at baseline or during the follow-up period, are asked to do a three-day cost registration and then a nutritional specialist will be involved. They will get follow-up specifically on their challenges.

Sleep

As many people with PD have challenges regarding sleep the PT always asks about this. If they have any challenges, the PT starts by sending over some web-based resources on e-mail that they can look at before further follow-up on this in the next conversation.

The PT sends over

- Recommendations for good sleep hygiene
- A sleep brochure on sleep and PD from the Norwegian Parkinson association (NPA)
- A link to the Norwegian Parkinson Associations resource page on sleep and PD
- A link to a podcast on sleep and PD

Activity wristband/watch

The PT follow up on the use of the wristband. Repetition on how to use it if needed. Based on the interest and capabilities of the individual participant the PT also gives an explanation on different activity goals on the wristband. How are they calculated, how can they be used as motivators for activity and how can you track your heartrate and use that to increase the intensity in exercise sessions.

It will not always be possible to discuss all these elements during the first conversation. This will vary depending on the individual participant. The PT keeps a short log of all conversations, noting themes that could be discussed, and elements to follow up on in the next conversation.

**Manual for follow-up conversations**

Time of conversation: once a month, to be agreed from time to time.

Conducted on teams/phone

Duration of call: Up to 60 min

You will not necessarily discuss all themes in this manual in each conversation, this must be individually adjusted. You should talk about exercise, and follow-up on specific goals or challenges in all conversations.

The PT can discuss and answer questions on all themes but should refer to other relevant health personnel in areas where they cannot or should not answer.

General

How are you doing? – The PT gives the participant space to say something without steering towards a specific theme.

Exercise

Which questions to ask will depend on the individual participant and situation, see examples below. The goal is to get the participants perspective and thoughts on progress, barriers and what is going well. Should something be changed, and how can it be done? If they present barriers the PT should support and guide them without making plans for them. The PT can make suggestions on how to adjust the plan if applicable, e.g., “you say you have troubles getting your heartrate up when you go for an exercise walk. What do you think about trying to add some more uphill walking during your walks to see if this can increase your heartrate?”.

- “Can you tell me about your exercise since last we spoke”
- “How do you feel that your exercise plan is working for you?”
- “Do you feel that your exercise plan is helping you reach your goals?”
  - “What do you think you have to do to reach your goals?”
- “What is you motivation for following the plan?”
- “Is there anything you feel you master well?”
- “Is there anything that is difficult?”
- “Do you find that you are able to push yourself when you exercise?”
- “Are you watching your heart rate? Are you training with a high heartrate? Is there anything you should or could do to exercise with a higher intensity?”
- “You had a plan to add more uphill to your daily walks to get a higher heartrate, how has that worked?”
- “You had a plan to test out going to spinning sessions, how has that worked?”
- “You planned to create a weekly exercise schedule and try to follow it? How has that worked?”
- “Do you have any questions or things you want to discuss regarding exercise?”

Activity wristband

Find out if they need any repetition on the use of the wristband or the tracking of activity.

Further questions must be adjusted to the individual based on how they use it, if they find it hard to use it or not, and how interested they are in using it and talking about it.

- “How is the use of the activity wristband going?”
- “Do you analyse you exercise/activity?”
- “Do you find it motivating? How?”
- “Do you have any questions or things you want to discuss regarding the activity wristband?”

Nutrition

Follow up on the potential specific challenges that have emerged in previous conversations. Follow up on specific plans for the nutritional group.

General questions that could be applicable for all, you must adjust this to the individual.

- “Do you experience any changes in weight?”
- “Do you experience any changes in appetite?”
- “If any nutritional goals or plans, how are the following up on this working?”
- “Do you have any questions or things you want to discuss regarding nutrition?”

Sleep

Map what the challenges regarding sleep are and ask what they have and have not tried and if they feel that something is working for their sleep issues.

Go through the basic sleep hygiene recommendations.

Give advice on their specific issues based on recommendations and guidelines. Refer to other health care professionals when applicable.

Medication

“Have you done any changes in medication?” Discuss this and answer questions if applicable, but the PT should not give specific advice on medication.

**Resources**

General resources

- Norwegian Parkinson Association: [Norges Parkinsonforbund](https://parkinson.no/)
- Fram Modellen: [FRAM MODELLEN (unicare.no)](https://unicare.no/content/uploads/Frammodellen-Parkinson-2021-1-2.pdf)
- ParkinsonNet international: [Home - ParkinsonNet International](https://www.parkinsonnet.com/)
- ParkinsonNet Norway: [ParkinsonNet - Helse Stavanger (helse-stavanger.no)](https://helse-stavanger.no/fag-og-forskning/kompetansetjenester/nasjonal-kompetansetjeneste-for-bevegelsesforstyrrelser/parkinsonnet)

Exercise resources

- Guidelines on exercise and Physiotherapy: [Physiotherapy (ENG, GER, POR, FIN, CZE) guideline - Parkinson's - ParkinsonNet International](https://www.parkinsonnet.com/discipline/physiotherapy/)
- Norwegian Parkinson Association: [Trening - Norges Parkinsonforbund](https://parkinson.no/behandling-og-rehabilitering/trening)

Nutrition resources

- Info page nutrition: [Mat og ernæring - Norges Parkinsonforbund](https://parkinson.no/behandling-og-rehabilitering/mat)
- Guidelines on nutrition: [Guideline Nutrition (ENG) - Parkinson's - ParkinsonNet International](https://www.parkinsonnet.com/discipline/nutrition/)
- Matvett på Nett: [Parkinson: Matvett på nett (parkinsonmatvett.vercel.app)](https://parkinsonmatvett.vercel.app/?fbclid=IwAR3YnNXMkptpa69ONcK5HgfFwXL6TlUxLdnnGg0flrOmrwIseBiYQmWAVHA)

Sleep resources

- Info page on sleep and PD: [Søvn - Norges Parkinsonforbund](https://parkinson.no/om-parkinson/symptomer/s%C3%B8vn)
- Podcast on sleep and PD: [Søvn – ParkisPodden – Podcast – Podtail](https://podtail.com/podcast/parkispodden/sovn/)
- Infopage the Norwegian centre for sleep, SOV.no: [Nasjonal kompetansetjeneste for søvnsykdommer (SOVno) - Helse Bergen (helse-bergen.no)](https://helse-bergen.no/nasjonal-kompetansetjeneste-for-sovnsykdommer-sovno)
